# Biological and Physiological Responses of Root-knot Disease Development on Five Cucurbits Exposed to Different Concentrations of Sulfur Dioxide

**DOI:** 10.3390/toxics11040334

**Published:** 2023-03-31

**Authors:** Tanveer Fatima Rizvi, Mujeebur Rahman Khan

**Affiliations:** Department of Plant Protection, Faculty of Agricultural Sciences, Aligarh Muslim University, Aligarh 202002, India; mrkhan.amu@gmail.com

**Keywords:** SO_2_ toxicity, cucurbits, intermittent exposures, root-knot, *Meloidogyne incognita*

## Abstract

A study was undertaken in order to investigate the effects of SO_2_ (25, 50, and 75 ppb) exposure for five hours on alternate days for three months on the susceptibility of five cucurbits to the infection of *Meloidogyne incognita*, causing root-knot disease. Four-week-old cucurbit plants were inoculated with 2000 J_2_ of *M. incognita*. SO_2_ levels of 50 and 75 ppb caused noticeable injury to foliage and reduced the plant growth parameters and biomass production of cucurbits (*p* ≤ 0.05). Nematode-inoculated plants caused characteristic oval, fleshy and large galls. The galls were formed closely, and as a result they coalesced, giving bead-like impressions especially in pumpkin and sponge gourds. Disease severity became aggravated on plants exposed to SO_2_ at 50 or 75 ppb concentrations. The nematode and SO_2_ interaction varied with the levels of SO_2_ and the response of the plant to *M. incognita*. SO_2_ at 50 or 75 ppb concentrations stimulated the pathogenesis of *M. incognita* on cucurbit species. The combined effect of 75 ppb SO_2_ and *M. incognita* suppressed plant length by 34% against the sum of decreases observed by *M. incognita* and SO_2_ individually (14–18%). At 50 ppb SO_2_, the fecundity of *M. incognita* was decreased and combined effect of SO_2_ and *M. incognita* was more than the sum of their singular effects. The study has proven that root-knot disease might become aggravated in the regions contaminated with elevated levels of SO_2_.

## 1. Introduction

The growth and survival of living beings mainly depend on air, which is a scarce commodity. Due to emissions from diversified industrialization and several other urban activities, the constitution of air’s small elements might change. Fossil fuels are still the prime energy source for running industry in developing countries, which results in high SO_2_ emission levels [1]. Amongst the most significant air pollutants, SO*_2_* is common in areas utilizing coal as a key source of energy [2,3]. The utilization and production of petroleum products are another major cause of SO_2_ liberation [4,5]. In developing countries like India, the chief source of SO_2_ production is thermal power plants, where an average of 125 million tons of coal is burnt per year [6]. These power plants are mostly located in rural areas, which means that a major portion of agricultural land is exposed to SO_2_ [7], leading to a negative effect on plant growth [8]. SO_2_ at 50–150 ppb (parts per billion) concentrations causes injury to green plants, depending on the species [9]. SO_2_ is a toxic gas, and causes distinguishing symptoms on leaves such as chlorosis, browning and yellowing [10]. Some studies have reported characteristic interveinal necrosis and the browning of leaves at 50 ppb SO_2_ [11].

As a consequence of injury caused by the gas, photosynthesis and other metabolic processes of plants are affected severely [12]. It is quite possible that SO_2_ may also affect the vulnerability of the plant to pathogens and/or its pathogenicity, if the infected plants are exposed to environments with higher concentrations of SO_2_ [7]. The above studies proved that SO_2_ exposures may influence the pathogenesis of phyto-nematodes, and the plants exposed may react differently to the pathogen [9]. Cucurbits are susceptible crops for root-knot nematode, and exhibit reduction in the yield [13]. The susceptibility of this crop to the pathogen may be influenced when plants are exposed to SO_2_ gas [14]. It is, therefore, important to study the effects of root-knot nematode on susceptible plants under elevated SO_2_ levels. The evidence available suggests that greater SO_2_ concentrations may have an impact on the development of plant diseases brought on by nematodes, despite the fact that few studies have been conducted to examine disease development under high SO_2_ levels. In the current study, root-knot disease induced by *M. incognita* on five cucurbits, including pumpkin, bitter gourd, bottle gourd, sponge gourd and cucumber, was evaluated in open-top chambers to determine the influence of SO_2_ relevant to the current and future anticipated ambient levels. Along with morphological factors, significant physiological, biochemical, and anatomical factors were also examined in connection with SO_2_ exposure and nematode inoculation (J_2_), including photosynthetic rate, transpiration rate, phenolic acid content, salicylic acid content, stomatal conductance, chlorophyll, and carotenoid contents.

## 2. Materials and Methods

### 2.1. Exposure System

**Figure 1:**
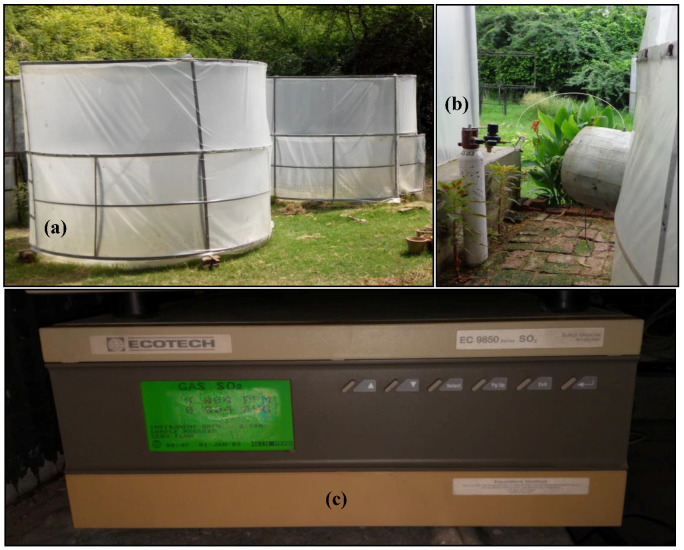
Open-top exposure chambers used to expose plants to SO_2_ (**a**); Exposure chamber attached to SO_2_ cylinder (**b**); SO_2_ gas analyzer (**c**).

Plants were subjected to SO_2_ exposure using 2 × 2.5 m (diameter × height) cylindrical open-top exposure chambers (OTCs; Figure 1). These OTCs were in dynamic condition, and no stagnation of air was observed in any area of the OTC [15]. The airflow within the chamber ranged between 0.2 and 0.5 m/s at various heights, and the air was completely replaced after every 1.0 to 1.5 min. As the chambers were made of transparent polythene sheets and had open tops, no significant changes in the incidence of sunlight were observed. In order to introduce the SO_2_ air mixture into the chamber, the inferior half of the chambers’ walls were made of two layers, and the internal layer of the wall was perforated. A gas cylinder containing a mixture of 50% SO_2_ and 50% N_2_ was used to introduce the SO_2_ gas into the OTCs (Sigma Gases, New Delhi, India). With the help of the SO_2_ gas analyzer, the amount of SO_2_ in the chamber was measured (EC9820 Ecotech, Melbourne, Australia; Figure 1).

To determine the SO_2_ level within the chamber, 3 Teflon input pipes were installed at heights of 1, 2, and 3 feet. A Teflon suction pump fed the air and gas combination to the SO_2_ gas analyzer. The SO_2_ content varied by 1 to 3 percent at different heights, according to the data. In order to reduce locational variability in the SO_2_ level, samples of SO_2_ were taken from the top three heights within the chamber during the exposure of the plants. In each chamber, SO_2_ levels were monitored once every 45 min for 5 min of sampling. To drain extra gas mixture, an exhaust pump was installed at the SO_2_ analyzer’s exhaust aperture. The rate of SO_2_ gas flow from the cylinder and the blowing assembly speed were controlled to maintain the desired level of SO_2_, which was 25, 50, and 75 ppb (5 h mean) [15].

Eight identically shaped exposure chambers were installed. In order to prevent the returning of the SO_2_–air mixture exhausted from the top, the chambers were positioned in a field with 7–8 m spacing. The area was located at 27°55′23.6″ N 78°04′29.2″ E, and the weather was mostly semi-arid [15]. Eight experimental treatments, i.e., four SO_2_ levels (ambient, 25, 50, and 75 ppb) with root-knot nematode inoculation, and the four SO_2_ level without nematode, were maintained. Two similar exposure chambers were used to expose the uninoculated control species (without *M. incognita*) and inoculated control species (with *M. incognita*) to ambient air (containing 3–5 ppb SO_2_). The rate of air flow and exposure period were also similar for both sets of plants. After the exposure, the pots were left within the chambers.

### 2.2. Isolation, Identification and Mass Culturing of Root-knot Nematode, Meloidogyne incognita

Infected root samples collected during the survey were gently washed and examined for the presence of egg masses and galls. *M. incognita* Kofoid and White was identified on the basis of perineal pattern [16]. Mature females were dissected from the galls, and perineal patterns were prepared and examined microscopically for the identification of *M. incognita*. Since root-knot nematode is an obligate parasite, its pure culture was maintained on eggplant, tomato and cucurbits. The pure culturing of *M. incognita* was initiated with single egg mass inoculation in eggplant, *Solanum melongena* L. cv. Pusa Purple Long in clay pots followed by the inoculation of eggplant seedlings, with the nematode culture emerging from the single egg mass inoculated plants. Mass culture of the nematode was maintained on perennial eggplant cv. Pusa Purple Round in a culture bed.

### 2.3. Inoculation with Root-Knot Nematode

Egg masses were excised from the eggplant’s roots, which were grown in the nematode culture bed. The egg masses were incubated on a wire gauge placed on a Petri plate with sufficient water at 30 °C for 7 days inside a BOD incubator. Nematode larvae (J_2_) hatched out from the eggs were collected and stored with water in a refrigerator. Three-week-old plants in pots were inoculated with the nematode suspension containing 2000 s stage juveniles of *M. incognita* plant.

### 2.4. Germplasm Collection and Plant Culture

The germplasm of five tested cucurbits viz., bottle gourd (*Lagenaria siceraria* (Mol.) Standl.), pumpkin (*Cucurbita pepo* L.), sponge gourd (*Luffa cylendrica* (L.) M. Roemd.), bitter gourd (*Momordica charantia* L.) and cucumber (*Cucumis sativus* L.) were purchased from the ICAR-Indian Institute of Vegetable Research, Varanasi, India. It was unknown how these cucurbits would react to SO_2_ exposure and/or infection with root-knot nematode. All of the seeds underwent 0.5% NaOCl surface sterilization. In clay pots with a 15 cm diameter, 4 seeds/pot were sown in a 3:1 autoclaved field soil and compost mixture. Seedlings were trimmed 4 weeks after sowing in order to have one seedling in each pot. Then, after, the pots were transferred into the OTCs and the nematode inoculation was carried out. For each treatment, five replicates were maintained. On alternate days, 250 mL of tap water was used to water the pots. Intermittent exposures started on alternate days (two days after the inoculation of cucurbits with root-knot nematode), which lasted for three months (total of 46 exposures of 5 h duration). Throughout the experiment, the plants were monitored regularly for symptoms of root-knot, SO_2_ injury, etc. The symptoms, soil population, number of galls and egg masses of root-knot nematode, gas injury, plant length, photosynthetic rate, transpiration rate, salicylic acid levels, stomatal conductance, total phenols, and leaf pigments were all measured at the time of harvest.

### 2.5. Soil Population, Number of Galls and Egg Masses of M. incognita

At harvest, roots were gently washed with water and then examined to count the number of galls and egg masses. Soil populations of the nematode were evaluated using Cobb’s decanting and sieving method [17].

### 2.6. Estimation of Chlorophyll and Carotenoid Contents

Total chlorophyll and carotenoids contents present in the leaves of the tested cucurbits were estimated at the time of harvest using the Lichtenthaler and Buschmann method [18]. A total of 1 g of fresh leaves from interveinal regions was ground in 80% acetone (40 mL) with the use of a mortar and pestle. After that, the solution was poured into a Buchner funnel covered with Whatman filter paper (No. 1). With the assistance of a suction pump, the filtration was completed. Acetone was added, and the residue was crushed thrice in it. In the Buchner funnel, the suspension was then transferred before being filtered under vacuum. Afterwards, the mortar and pestle were washed with the acetone and suspension was poured into the funnel for filtration. In a volumetric flask measuring 100 mL, the filtrate was decanted. Acetone was added to bring the volume up to the capacity. A UV spectrophotometer (Shimadzu, Japan) was used to measure the optical density (OD) of the filtrate at 470 nm for carotenoids and at 645 and 662 nm for total chlorophyll contents [18].

### 2.7. Determination of Number of Trichomes and Stomata

Fully grown and fresh leaves (2–3 leaves per plant) of roughly the same size and age were preserved in 70% ethyl alcohol after being fixed in formalin aceto-alcohol (FAA). To remove the epidermal peels, 1 cm^2^ pieces of the leaves were then cut into pieces and boiled in HNO_3_ (4%) [19]. After being rinsed with tap water, the peels were stained with hematoxylin and iron-alum. The peels were mounted in Canada balsam for microscopic inspection after being dehydrated in alcohol series. An assessment of the number of stomata and trichomes/cm^2^ peel was calculated.

### 2.8. Estimation of Foliar Phenolic Compounds and Salicylic Acid Contents

Leaves of cucurbit species were taken at the time of harvest from each treatment and chopped into pieces that ranged in size from 0.5 to 1.0 cm. Then, pieces of 1 g were soaked in water for one night. Whatman filter paper (No. 1) was used to filter the suspension. After completing the ethyl acetate fraction, the water content was removed using Na_2_SO_4_ and the solution was then evaporated with the help of a water bath. The dried sample was mixed with 10 mL of methanol, and the suspension was then used to measure the absorbance at 306 nm with a spectrophotometer (UV 2450, Shimadzu, Japan). Salicylic acid levels of 0, 10, 20, 30, 40, 50, and 100 ppb in methanol were used to prepare the salicylic acid standard curve. The formula y = mx c was used to calculate the salicylic acid concentration in the sample based on the standard curve [20].

A modified Folin–Ciocalteu technique was used to assess the total phenolic contents of each leaf [21]. The filtrate was diluted through distilled water (0.4 mg/5 mL). Folin–Ciocalteu reagent (2.5 mL, 1/10) was mixed with the diluted solution (0.5 mL). After allowing the mixture to sit at room temperature for two minutes, an aqueous solution of sodium carbonate (2 mL; 75 g/L) was added to the solution. The mixture was completely blended, incubated for 15 min at 50 °C, and allowed to cool in an ice bath. A blank was made using only distilled water. The absorbances were determined using a UV spectrophotometer at 760 nm (Shimadzu, Japan). Then, 1–8 µg/mL of Gallic acid was used for the preparation of a calibration curve. According to the calibration curve, y = 0.133x + 0.001 (r2 = 0.999), where x specifies the concentration of Gallic acid in mg/L and y denotes the absorbance. The total phenolics were estimated in mg of Gallic acid equivalents/g of dry extract [21].

### 2.9. Physiological Parameters

The stomatal conductance, photosynthetic rate, and transpiration rate were evaluated using the portable photosynthesis analyzer (LI625XT, Li Cor, Lincoln, NE, USA) and Fluorescence Analyzer (PAM, Walz, Germany). These variables are essential for determining the overall effects of SO_2_ on the physiology of a plant [15].

### 2.10. Statistical Analysis

To evaluate the reproducibility of the results, experimentations with identical treatments were repeated for 2 years. At *p* ≤ 0.05, it was determined that the effects of nematode infestation and SO_2_ levels on the study’s variables were statistically identical across 2 years. Therefore, the 2 years’ data were pooled. For each treatment, the mean of 5 replicates/year (10 replicates) were evaluated and represented in tables and figures. A 2-factor analysis of variance was applied to the data (10 repetitions). The SO_2_ concentrations (4 treatments, 3–5, 25, 50, and 75 ppb) were treated as factor one, and the root-knot nematode inoculation as factor two (2 treatments). The Tukey honest significant difference test was applied for pair-wise comparison of contrasts related to SO_2_ levels and their interactions with nematodes. Whole analysis was carried out using the “R” software package [22]. Using the Agricolae package of R software, LSD was evaluated to ascertain significant treatments at probability levels of *p* ≤ 0.05. The data on plant length, photosynthetic rate, transpiration rate, salicylic acid and total phenolic content, stomatal conductance, chlorophyll and carotenoids content, and number of trichomes and stomata were subjected to the 3-factor analysis. By using a 2-factor ANOVA analysis, the impact of SO_2_ levels on disease severity, nematode population, number of galls, and fecundity were evaluated. Significant treatments were found at probability levels of *p* ≤ 0.05 [23]. The percentage increase or decrease in comparison to the control was evaluated and used to explain the results.

## 3. Results

### 3.1. Symptoms

**Figure 2:**
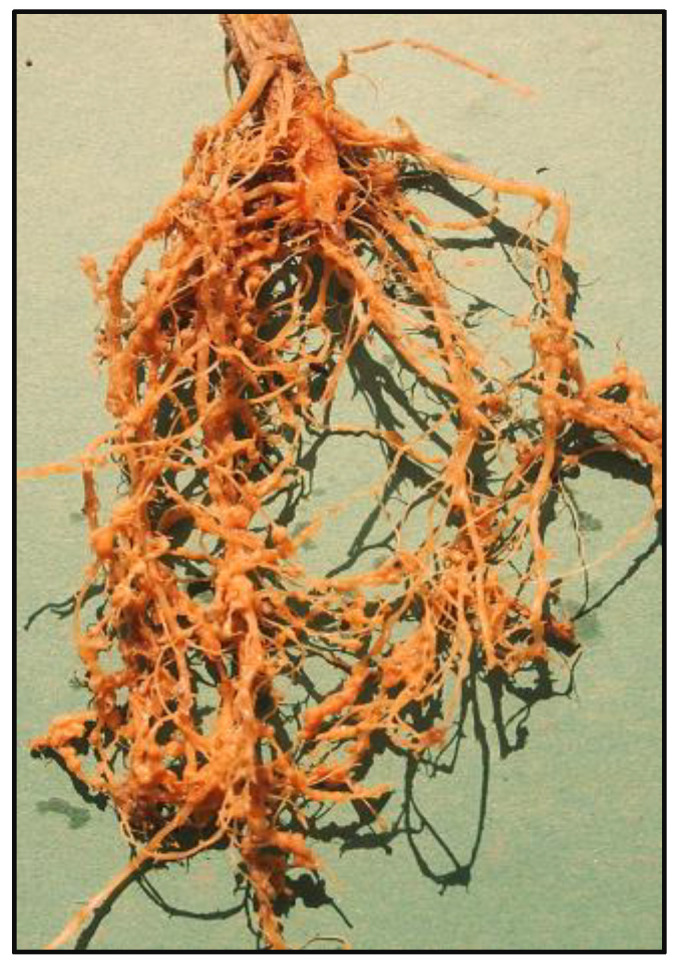
Galling on roots of cucurbit species caused by root-knot nematode.

Inoculation with 2000 J_2_ of *M. incognita* caused characteristic oval, fleshy and large galls on the roots of cucurbits (Figure 2). The galls were formed closely, and as a result they coalesced, giving bead-like impressions, especially in pumpkin and sponge gourds. The bitter gourd was found almost resistant to the nematodes, as only a few galls were formed on this crop. The order of severity of the root-knot disease in terms of number of galls and egg masses in tested cucurbits evaluated was pumpkin (46%) > bottle gourd (43%), sponge gourd (43%) > cucumber (39%) > bitter gourd (18%).

**Figure 3:**
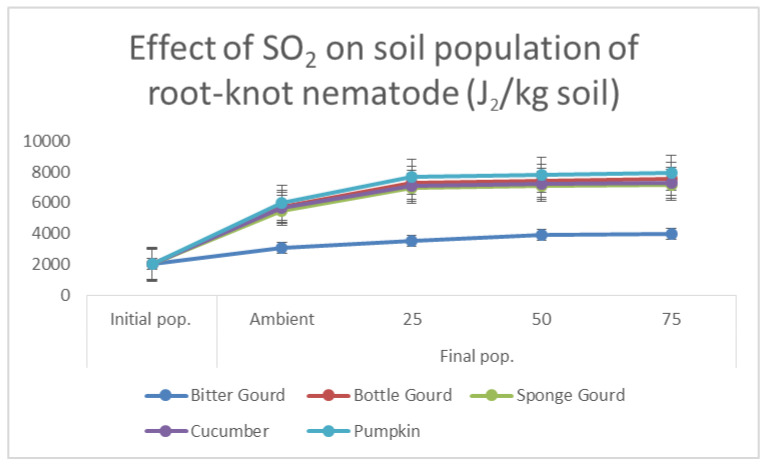
Line diagram, showing the effect of SO_2_ exposure on soil population of root-knot nematode, in five cucurbits.

The exposure of plants to SO_2_ promoted galling in all the cucurbits. A significant increase in the number of galls/root system was recorded at 50 and 75 ppb for exposed plants. The egg mass production was also significantly increased at this concentration. The soil population of the nematode was significantly increased in 50 and 75 ppb SO_2_-exposed plants (Figure 3). The bitter gourd produced negligible galls, as it had some resistance against the nematode. However, the exposure of SO_2_ broke the resistance in bitter gourd, especially at 50 and 75 ppb concentrations. The order of susceptibility of the cucurbits to *M. incognita* was pumpkin > bottle gourd > sponge gourd > cucumber > bitter gourd.

**Figure 4:**
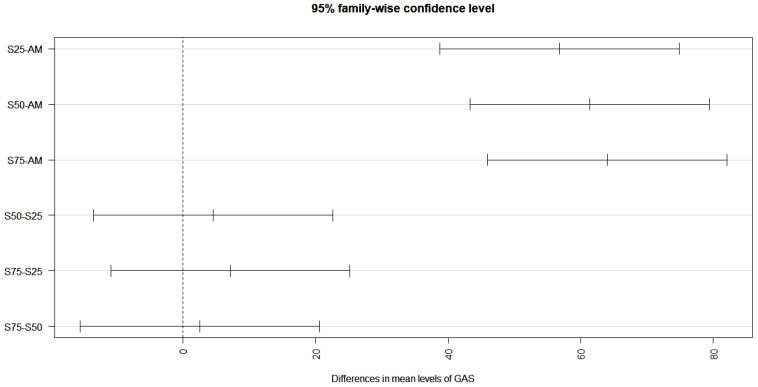
Effect of SO_2_ levels on galling of M. incognita in pumpkin. The contrasts located on the right side of the line of no difference represent positive interaction (synergistic). The contrasts situated at the furthest location show highest interaction effect.

The orthogonal contrasts prepared between SO_2_ levels and galling on pumpkin showed mostly significant interactions between SO_2_ and *M. incognita*, as only a few contrasts lay on the broken vertical line of no difference. Maximum synergistic interaction with regard to an increase in the galling was observed at 75 ppb SO_2_, as this contrast was situated at the furthest location from the line of no difference (Figure 4). The interactive and individual effects of SO_2_, crops and *M. incognita* were significant at *p* ≤ 0.001 (Table 1).

### 3.2. Gas Injury

**Figure 5:**
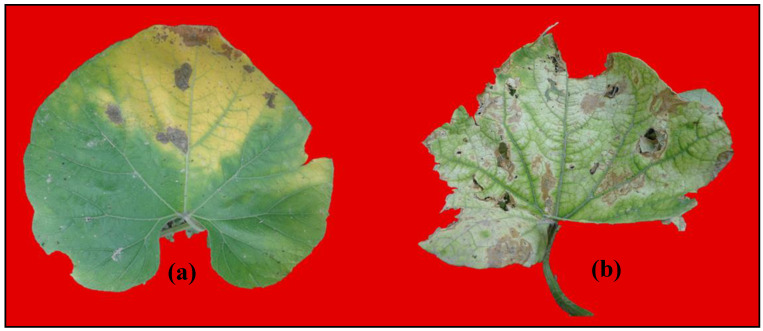
Gas injury caused by SO_2_ exposure at (**a**) 50 ppb and (**b**) 75 ppb.

The five cucurbits, viz., bitter gourd, bottle gourd, cucumber, pumpkin, and sponge gourd tested in the study were found to be sensitive to 50–75 ppb SO_2_ and developed characteristic marginal and interveinal chlorosis and browning, especially at 75 ppb concentration (Figure 5).

### 3.3. Plant Growth Parameters

**Figure 6:**
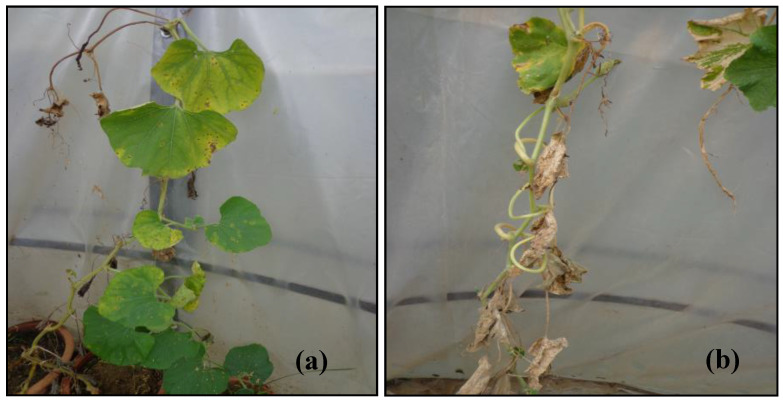
Gas injury caused by SO_2_ exposure in inoculated plants at (**a**) 50 ppb and (**b**) 75 ppb.

**Figure 7:**
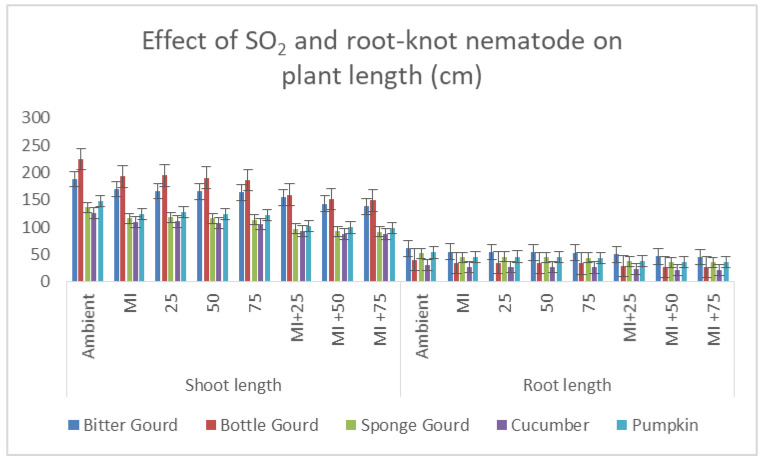
Bar diagram showing the effect of SO_2_ exposure and root-knot nematode on the plant length of five cucurbits.

Intermittent exposures of SO_2_ decreased the plant length and biomass production of plants. The decrease in the plant growth parameters was significant for all tested cucurbit species, with relatively greater damage to pumpkin and sponge gourd at 50 and 75 ppb SO_2_ in comparison to 25 ppb SO_2_ or control (ambient air, 3–5 ppb SO_2_). In addition, all species of cucurbits used in experiment, with the exception of the bitter gourd, have seen a substantial reduction in length and dry weight (shoot) after inoculation with root-knot nematode in comparison to the uninoculated control (*p* ≤ 0.05; Figure 6; Table 2). However, in plants inoculated with root-knot nematode, the dry weight of the root increased to some extent due to the formation of galls and egg masses. The nematode’s individual impacts on the aforementioned two variables were shown to be significant by ANOVA at *p* ≤ 0.001. A major decrease in the plant growth was recorded in concomitantly inoculated and exposed cucurbit plants as compared to the control. The singular effects of SO_2_ at *p* ≤ 0.05 on the plant growth parameters and root-knot nematode were significant at *p* ≤ 0.001. Overall, the order of decrease in plant growth parameters was pumpkin > sponge gourd > bottle gourd and cucumber > bitter gourd, as compared to the respective control (Figure 7).

The interaction between M. incognita and 75 ppb SO_2_ was found to be synergistic, resulting in a reduction in plant height of more than the addition of decrease caused by 75 ppb SO_2_ and nematode singly. For example, 75 ppb SO_2_ decreased the plant height by 18%, whereas M. incognita inoculation decreased plant height by 14%; the sum is 32%. The combined treatment of M. incognita with 75 ppb SO_2_ led to a 34% reduction in plant length (Figure 7). Although this was 2% greater than the sum of individual effects (synergistic interaction), statistically it is not significant at *p* ≤ 0.05. Hence, the interaction of the nematode at 75 ppb SO_2_ was statistically additive for most of the plant growth variables (Figure 7). However, a nearly additive interaction was recorded for 25 ppb SO_2_ and the nematode.

### 3.4. Number of Trichome and Stomata

SO_2_ exposures induced some modification on the epidermal characteristics of cucurbit leaves. The 50 and 75 ppb SO_2_ concentrations decreased the frequency of trichomes and stomata per unit leaf surface over the control (Table 3), whereas in nematode-infected plants these epidermal characters were not significant; in concomitantly exposed and inoculated plants, the frequency of trichomes and stomata decreased compared to the respective control. Overall, the stomatal density and the number of trichomes on the surface of the leaves in plants with the nematode inoculation were lower, significantly, than in uninoculated plants in the SO_2_ treatments (*p* ≤ 0.05).

In exposed and inoculated plants, the density of the stomata and trichome decreased significantly and the maximum decrease was observed at the 75 ppb concentration of SO_2_ (19–32%), followed by 50 (18–30%), 25 (9–11%), and ambient concentrations (Table 3).

### 3.5. Foliar Pigments, Salicylic Acid Contents and Phenolic Compounds

**Figure 8:**
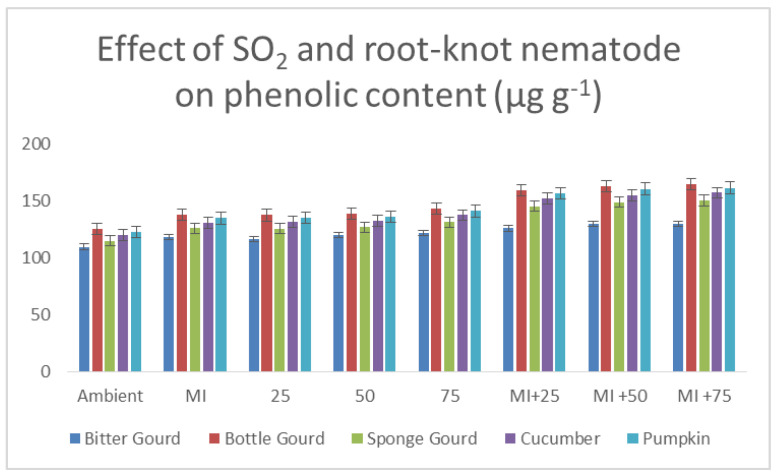
Bar diagram showing the effect of SO_2_ exposure and root-knot nematode on the total phenolic content of leaves of five cucurbits.

Alternating the exposures of 50 ppb SO_2_ to plants caused in a significant rise in TPC (total phenolic content) and SAC (salicylic acid contents) of the leaf as compared to 25 ppb SO_2_ or the respective control (*p* ≤ 0.05; Table 4). Inoculation with M. incognita caused a significant rise (*p* ≤ 0.05) in SAC and TPC for all cucurbits species used in the experiment when compared to the control (9–11%; *p* ≤ 0.05; Table 4; Figure 8). The cumulative effects of SO_2_ and nematode on SAC and TPC were almost equal to the total increase in SAC and TPC caused by M. incognita and SO_2_ separately. The effects of SO_2_ and the nematode on the SAC were determined to be significant at *p* ≤ 0.001 (Table 4).

**Figure 9:**
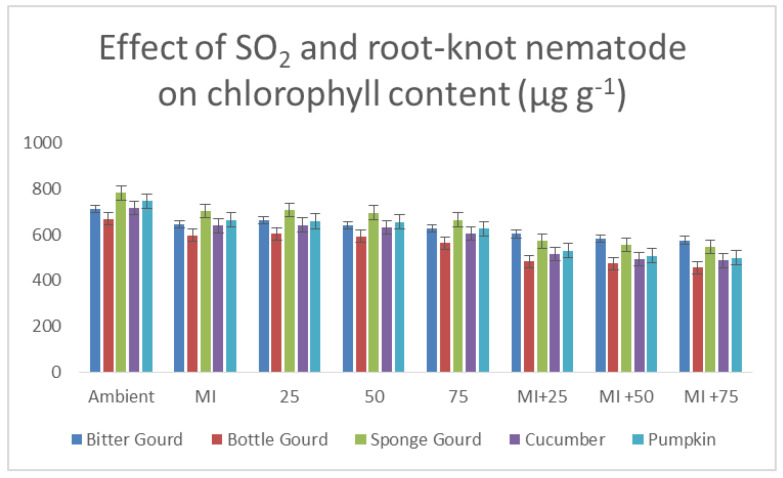
Bar diagram showing the effect of SO_2_ exposure and root-knot nematode inoculation on the total chlorophyll content of leaves of five cucurbits.

Chlorophyll content decreased with the increasing SO_2_ levels, and the maximum decrease was recorded at 75 ppb SO_2_ (Figure 9). When exposed to 50 and 75 ppb SO_2_, the contents of leaf carotenoids were reduced in comparison to the corresponding controls (Table 4). Inoculation with the nematode resulted in a significant decrease in chlorophyll content and carotenoids in all tested cucurbits in comparison to the respective controls. The sequence of the decrease in chlorophyll and carotenoid contents was: pumpkin > cucumber > bottle gourd > sponge gourd > bitter gourd. Chlorophyll and carotenoid contents in inoculated as well as exposed plants (50 or 75 ppb SO_2_) exhibited a further decrease when compared to nematode-injected corresponding controls (10–21%; Table 4; Figure 9). ANOVA revealed that the individual and combined effects of SO_2_ and the nematode were significant for each at *p* ≤ 0.001. 

### 3.6. Rate of Photosynthesis and Transpiration and Stomatal Conductance

**Figure 10:**
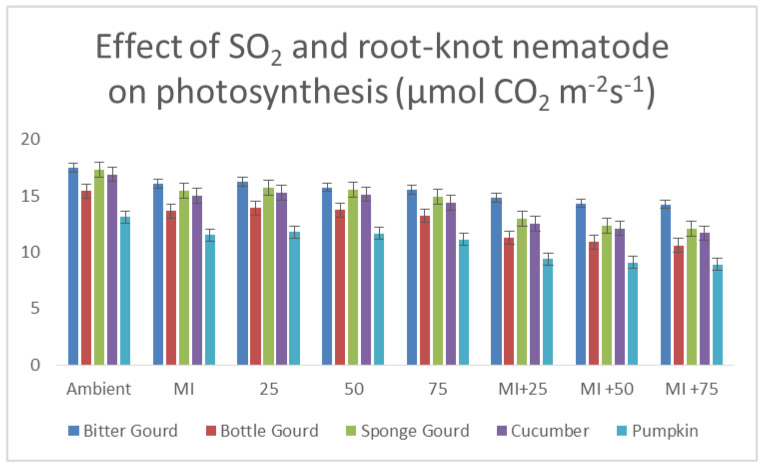
Bar diagram showing the effect of SO_2_ exposure and root-knot nematode inoculation on the rate of photosynthesis of five cucurbits.

With increasing SO_2_ levels, the photosynthesis rate of inoculated plants dropped significantly (Figure 10). The lowest photosynthetic rate was reported as 11–15% lower than the control at 75 ppb SO_2_. The photosynthesis rate at 50 ppb SO_2_ was reduced by 9–11%, i.e., 4–5% lesser than 75 ppb, but significantly lower than at 25 ppb SO_2_ and ambient control. The difference between 25 ppb SO_2_ and ambient control (3–5 ppb) was negligible. However, in contrast to the ambient control, the transpiration rate and stomatal conductance considerably increased with increasing SO_2_ levels (Table 5). According to statistics, the trend in the rise in stomatal conductance or transpiration rate was more or less equivalent to the decrease in photosynthetic rate at *p* ≤ 0.05. Comparing plants that had been infected with M. incognita to those that had not indicated a substantial reduction in the rate of photosynthetic activity, and increases in the rate of transpiration and stomatal conductance were observed as well. The order of the different cucurbits’ decreased photosynthetic rates and increased transpiration rates as a result of root-knot disease was: pumpkin > bottle gourd > sponge gourd > cucumber > bitter gourd (Figure 10; Table 5). The stomatal conductance’s response pattern and rate of transpiration were statistically identical.

The physiological factors of cucurbits responded to intermittent inoculation and exposure in a manner that was mostly consistent with the effects seen from M. incognita and SO_2_ levels individually. In comparison to the nematode-inoculated control, the photosynthesis rate in the nematode plus SO_2_ treatments reduced with increasing amounts of SO_2_ (25–75 ppb), but it was higher considerably than that in the SO_2_ exposed plants without inoculation of the nematode (*p* ≤ 0.05). Likewise, the stomatal conductance and transpiration rates also increased further with the increasing SO_2_ levels in inoculated and exposed plants.

## 4. Discussion

The cucurbit plants inoculated with second stage juvenile of *M. incognita* were found to be susceptible to the nematode and established distinctive symptoms of root-knot [24]. However, the bitter gourd showed lesser vulnerability to root-knot nematode damage. The infection process of *Meloidogyne* species in susceptible hosts started with the oriented movement of the juveniles through the chemical stimuli emanated in the form of root exudates. After approaching the root, the nematode juvenile started making perforations on the surface to enter into the roots of a susceptible plant species/cultivar [25]. On the other hand, the nematode-repellent substances present in the roots of non-host/immune plants opposed the nematode invasion. Hence, interactions between plants and nematodes could take place practically even before the nematode reached the plant roots [26]. The single-resistance dominant genes from plant interaction related to avirulence (Avr) genes in nematodes created an incompatible association, in contrast to compatible plant-nematode associations that took place in susceptible hosts [27]. This unfavorable association initiated a chain of plant reactions to counter the nematode-defense mechanisms [28]. As the *Meloidogyne* juveniles entered the plant roots, the Avr genes of the nematode produced effectors that caused the development and expression of Mi-resistant genes in plants, resulting in a sort of hypersensitive reaction [29]. The guard hypothesis is the second host-specific defense’s strategy, where nematode effectors first activate the plant virulence factors, which then activate the R-gene [30]. In addition, there are various other mechanisms of resistance, and thus the host defense depends on the activation of many identified and unidentified R-genes [31,32,33]. The symptoms of root-knot first appeared on the roots, and later galls could be seen in nodal and intermodal areas of the stem [25]. One of the most vulnerable hosts for *M. incognita* are cucurbit plants, and the development of galls, particularly on the roots, is a characteristic sign that the nematode has infected the plant. However, among the five different types of cucurbits, the disease severity varied considerably. According to reports, even though *M. incognita* is a pathogen of the majority of cucurbits, the relative vulnerability of each genus varies widely, and bitter gourd exhibits lower susceptibility than other cucurbits, as seen in the current study.

Root-knot nematode inoculation caused a substantial decrease in photosynthesis, leaf pigmentation, and plant development, as well as a rise in salicylic acid and phenolic content, the rate of transpiration and stomatal conductance, as compared to the control. The growth of the galls on the roots would have interfered with the plants’ ability to absorb water and nutrients, resulting in a sharp decline in leaf chlorophyll and carotenoids [26]. The effectiveness of the leaf’s photosynthetic process is closely associated with the reduction in photosynthetic pigments. As a result, all of the five infected cucurbit species showed a noticeably lower rate of photosynthetic activity. Additionally, the rate of photosynthesis is inversely related to the rates of transpiration and stomatal conductance [27]. Furthermore, the nematode infestation would have resulted in the partial closure of a stomata cavity or aperture. As a result, the rate of transpiration in the nematode-infected plants consistently dropped. The formation of galls on the root hindered the uptake of nutrients, minerals and water, subsequently resulting in poor productivity of photosynthate. The biochemicals that initiate host defense are salicylic acid and phenol, and their production is enhanced in response to any stress (biotic or abiotic) [28]. According to the results of the present investigation, pathogen and the gaseous exposures are among the crucial biotic stresses that can trigger the production of phenolic and salicylic acid components.

The rates of photosynthesis, leaf pigments, and plant development of cucurbit species were significantly reduced by exposure to 50 and 75 ppb SO_2_, but phenolic and salicylic acid content increased significantly. The gas entered plants by diffusion through the stomata [29], and produced sulfite ions which reacted with water molecules [3] (EPA 2021). These ions were slowly oxidized to sulfite and sulfate ions [30]. These ions, especially sulfites, are phytotoxic when found in excess in plants, and cause visible injury in green plants when they are exposed to above 50 ppb SO_2_ concentrations [7]. Plants exhibit disorders with specific symptoms due to SO_2_ exposure [31]. Chlorosis and necrosis (browning) or yellowing, which was established on the pumpkin foliage, may be due to the bleaching and/or photo oxidation of leaf pigments [30]. Under glasshouse conditions, 75 ppb SO_2_ suppressed mustard yield by 9–22% [7].

The cucurbit plants were susceptible to *M. incognita* infection, and intermittent SO_2_ exposures further predisposed host plants, resulting in altered host–pathogen interactions [32]. Interactions are additive when their combined effect is the sum of each independently, synergistic when the combined effect is greater than the sum of each independently, and antagonistic when the combined effect is less than the sum of each independently [33]. Most of the effects of nematode infection and gas exposures (especially mixtures) were synergistic [32]. In the present study, an additive interaction between the root-knot nematode and 25 ppb SO_2_ was recorded. However, the interaction between higher concentrations, especially 75 ppb and the nematode, was mostly synergistic because these concentrations aggravated the infection of *M. incognita* in all cucurbit species used in the experiment. Similar interactions were observed in a previous study, as well [30,31,32].

The bitter gourd had much fewer galls and egg masses, but when the plants were exposed to 50–75 ppb the severity of the disease in terms of number of galls and egg masses enhanced significantly, representing that the moderately resistant bitter gourd was altered into a host susceptible to *M. incognita*. The nematode’s juvenile entered the surface, as it is an endoparasite [34]. The present study revealed that, under 50–75 ppb SO_2_ exposures, the cucurbits’ plant growth may be reduced significantly and, meanwhile, plants can become more vulnerable to *M. incognita*, increasing the likelihood that the disease would spread widely, as was observed for all cucurbit plants used in the current study.

## 5. Conclusions

The study has revealed that increased SO_2_ concentrations on cucurbitaceous plants may have an impact on the host–parasite interaction of the root-knot nematode. Five different cucurbit species were evaluated, and intermittent exposure to SO_2_ at 50 and 75 ppb decreased plant growth parameters and biomass production up to 18%. The cucurbits’ susceptibility to *M. incognita* was further increased by SO_2_ exposures, resulting in an increase in root-knot disease severity and a decrease in plant growth parameters (34% at 75 ppb). Bitter gourd showed considerable resistance to nematodes; however, when the plants were exposed to 50–75 ppb SO_2_, the severity of the root-knot disease increased significantly (26–29%). A synergistic interaction between the root-knot nematode and 75 ppb SO_2_ was recorded, which is really concerning because concentrations of 25 ppb or more are relevant to current ambient SO_2_ concentrations. Environments near coal-fired thermal power plants, busy motorways, oil refineries, and other anthropogenic sources may have higher levels of SO_2_, even as high as 50 ppb.

## Figures and Tables

**Table 1:** Effect of intermittent exposures of SO_2_ (ambient, 25, 50, and 75 ppb) on the number of galls and egg masses caused by root-knot nematode (*M. incognita*) on the roots of five cucurbits.

Cucurbits	Bitter Gourd		Bottle Gourd		Sponge Gourd		Cucumber		Pumpkin	
Treatments	No. of Galls/ Root	No. of Egg Masses/ Root	No. of Galls/ Root	No. of Egg Masses/ Root	No. of Galls/ Root	No. of Egg Masses/ Root	No. of Galls/ Root	No. of Egg Masses/ Root	No. of Galls/ Root	No. of Egg Masses/ Root
Ambient (Inoculated)	11.19 ^c^	5.15 ^c^	171.44 ^b^	99.31 ^b^	185.33 ^b^	106.33 ^b^	165.51 ^b^	92.11 ^b^	199.33 ^b^	110.03 ^b^
MI + 25	12.87 ^b^	5.94 ^b^	218.07 ^a^	126.12 ^a^	235.00 ^a^	134.83 ^a^	208.87 ^a^	116.06 ^a^	256.14 ^a^	141.06 ^a^
MI + 50	14.16 ^a^	6.53 ^a^	221.50 ^a^	128.21 ^a^	238.52 ^a^	136.74 ^a^	211.03 ^a^	117.35 ^a^	260.72 ^a^	143.48 ^a^
MI + 75	14.46 ^a^	6.66 ^a^	224.76 ^a^	130.39 ^a^	241.30 ^a^	138.87 ^a^	215.16 ^a^	119.47 ^a^	263.31 ^a^	145.24 ^a^
LSD (*p* ≤ 0.05)										
SO_2_ Level: Treatment	0.72	0.33	10.96	6.35	11.79	6.77	10.93	6.07	12.85	7.08

Ambient = 3–5 ppb SO_2_, i.e., air without any addition of SO_2_, MI = inoculation of plants soil with 2000 J_2_ of *M. incognita* plant. Figures in a column followed by different alphabets are significantly different at *p* ≤ 0.05 according to Tukey’s test.

**Table 2:** Effect of intermittent exposures of SO_2_ (ambient, 25, 50, and 75 ppb) and root-knot disease (*M. incognita*) on plant biomass production in terms of dry weight of the five cucurbits.

Cucurbits	Bitter Gourd		Bottle Gourd		Sponge Gourd		Cucumber		Pumpkin	
Treatments	Shoot	Root	Shoot	Root	Shoot	Root	Shoot	Root	Shoot	Root
Ambient (Uninoculated)	29.11 ^a^	10.06 ^a^	31.31 ^a^	9.31 ^c^	28.33 ^a^	9.83 ^a^	27.31 ^a^	9.09 ^c^	31.61 ^a^	10.71 ^ab^
25	25.68 ^bc^	8.85 ^bc^	27.24 ^b^	8.06 ^d^	24.68 ^b^	8.55 ^c^	23.81 ^b^	7.93 ^d^	27.12 ^b^	9.20 ^bc^
50	25.62 ^bc^	8.84 ^bc^	26.43 ^b^	7.87 ^d^	24.08 ^b^	8.38 ^c^	23.38 ^b^	7.79 ^d^	26.36 ^b^	8.92 ^c^
75	25.35 ^bc^	8.75 ^c^	26.02 ^b^	7.73 ^d^	23.66 ^b^	8.22 ^c^	22.89 ^b^	7.64 ^d^	25.89 ^b^	8.80 ^c^
Ambient (Inoculated)	26.20 ^b^	9.07 ^bc^	26.83 ^b^	7.97 ^d^	24.19 ^b^	8.39 ^c^	23.49 ^b^	7.84 ^d^	26.55 ^b^	8.97 ^c^
MI + 25	23.81 ^cd^	10.13 ^bc^	22.14 ^c^	10.21 ^b^	20.28 ^c^	10.03 ^b^	20.07 ^c^	9.73 ^bc^	21.81 ^c^	11.24 ^ab^
MI + 50	22.07 ^de^	10.55 ^bc^	21.13 ^c^	10.87 ^ab^	19.29 ^c^	10.71 ^b^	18.95 ^c^	10.32 ^ab^	21.12 ^c^	11.66 ^a^
MI + 75	20.84 ^e^	10.94 ^ab^	20.82 ^c^	11.23 ^a^	19.07 ^c^	10.91 ^ab^	18.79 ^c^	10.97 ^a^	20.86 ^cd^	11.94 ^a^
LSD (*p* ≤ 0.05)										
Treatment	0.59	0.22	0.61	0.06	0.55	0.19	0.53	0.18	0.6	0.2
SO_2_ Level	0.83	0.31	0.86	0.09	0.78	0.27	0.76	0.25	0.85	0.29
SO_2_ Level: Treatment	1.19	0.43	1.21	0.12	1.1	0.38	1.07	0.36	1.2	0.4

Ambient = 3–5 ppb SO_2_, i.e., air without any addition of SO_2_, MI = inoculation of plants soil with 2000 J_2_ of *M. incognita*/plant. Figures in a column followed by different alphabets are significantly different at *p* ≤ 0.05 according to Tukey’s test.

**Table 3:** Effect of intermittent exposures of SO_2_ (ambient, 25, 50, and 75 ppb) and root-knot disease on number of trichomes and stomata in five cucurbits.

Cucurbits	Bitter Gourd		Bottle Gourd		Sponge Gourd		Cucumber		Pumpkin	
Treatments	Trichomes (cm^−2^)	Stomata (cm^−2^)	Trichomes (cm^−2^)	Stomata (cm^−2^)	Trichomes (cm^−2^)	Stomata (cm^−2^)	Trichomes (cm^−2^)	Stomata (cm^−2^)	Trichomes (cm^−2^)	Stomata (cm^−2^)
Ambient (Uninoculated)	120.31 ^a^	88.11 ^a^	129.31 ^a^	93.21 ^a^	126.11 ^a^	84.33 ^a^	127.61 ^a^	98.33 ^a^	121.11 ^a^	92.22 ^a^
25	112.01 ^b^	82.12 ^b^	115.34 ^b^	83.24 ^b^	112.87 ^b^	75.64 ^b^	115.10 ^b^	88.79 ^b^	107.79 ^b^	81.80 ^b^
50	108.28 ^bc^	79.39 ^bc^	114.31 ^b^	82.49 ^b^	111.86 ^b^	74.80 ^b^	113.57 ^b^	87.71 ^b^	106.70 ^b^	81.15 ^b^
75	106.11 ^bcd^	78.24 ^bc^	108.75 ^b^	78.48 ^b^	106.69 ^b^	71.17 ^b^	108.47 ^b^	83.68 ^b^	101.13 ^b^	77.19 ^bc^
Ambient (Inoculated)	108.52 ^bc^	79.56 ^bc^	115.09 ^b^	82.77 ^b^	112.36 ^b^	75.14 ^b^	114.34 ^b^	87.91 ^b^	107.06 ^b^	81.61 ^b^
MI + 25	101.30 ^cde^	74.19 ^cd^	94.40 ^c^	68.23 ^c^	92.44 ^c^	62.07 ^c^	94.43 ^c^	72.37 ^c^	86.47 ^c^	66.03 ^c^
MI + 50	98.65 ^de^	72.16 ^d^	90.52 ^c^	65.06 ^c^	89.92 ^c^	59.96 ^c^	93.16 ^c^	71.19 ^c^	83.69 ^c^	63.91 ^c^
MI + 75	97.21 ^e^	71.02 ^d^	88.06 ^c^	63.38 ^c^	86.26 ^c^	58.02 ^c^	89.33 ^cd^	68.54 ^cd^	81.51 ^c^	61.97 ^cd^
LSD (*p* ≤ 0.05)										
Treatment	2.52	1.86	2.56	1.85	2.5	1.68	2.55	1.97	2.38	1.81
SO_2_ Level	3.58	2.62	3.62	2.61	3.54	2.37	3.61	2.78	3.37	2.56
SO_2_ Level: Treatment	5.06	3.71	5.11	3.69	5.01	3.35	5.11	3.93	4.76	3.63

Ambient = 3–5 ppb SO_2_, i.e., air without any addition of SO_2_, MI = inoculation of plants soil with 2000 J_2_ of *M. incognita*/plant. Figures in a column followed by different alphabets are significantly different at *p* ≤ 0.05 according to Tukey’s test.

**Table 4:** Effect of intermittent exposures of SO_2_ (ambient, 25, 50 and 75 ppb) and root-knot disease on salicylic acid content and carotenoids in five cucurbits.

Cucurbits	Bitter Gourd		Bottle Gourd		Sponge Gourd		Cucumber		Pumpkin	
Treatments	Salicylic Acid	Carotenoids	Salicylic Acid	Carotenoids	Salicylic Acid	Carotenoids	Salicylic Acid	Carotenoids	Salicylic Acid	Carotenoids
Ambient (Uninoculated)	16.31 ^c^	34.51 ^a^	18.31 ^c^	35.21 ^a^	15.11 ^c^	41.51 ^a^	17.11 ^c^	35.12 ^a^	18.91 ^c^	38.12 ^a^
25	17.35 ^b^	32.20 ^b^	20.10 ^b^	31.79 ^b^	16.47 ^b^	37.61 ^b^	18.74 ^b^	31.50 ^b^	20.82 ^b^	33.62 ^b^
50	17.86 ^b^	30.99 ^bc^	20.29 ^b^	31.20 ^b^	16.64 ^b^	36.99 ^b^	18.92 ^b^	31.05 ^b^	20.99 ^b^	33.51 ^b^
75	18.10 ^b^	30.23 ^bcd^	21.00 ^b^	29.65 ^b^	17.23 ^b^	35.20 ^b^	19.64 ^b^	29.71 ^bc^	21.75 ^b^	32.06 ^b^
Ambient (Inoculated)	17.66 ^b^	31.34 ^bc^	20.12 ^b^	31.58 ^b^	16.52 ^b^	37.40 ^b^	18.60 ^b^	31.47 ^b^	20.80 ^b^	33.85 ^b^
MI + 25	18.81 ^a^	29.26 ^cd^	23.24 ^ab^	25.60 ^c^	19.13 ^a^	30.30 ^c^	21.71 ^a^	25.29 ^c^	24.24 ^a^	27.14 ^c^
MI + 50	19.25 ^a^	28.30 ^d^	23.84 ^ab^	24.37 ^c^	19.52 ^a^	29.47 ^c^	22.23 ^a^	24.27 ^c^	24.77 ^a^	25.81 ^c^
MI + 75	19.29 ^a^	27.99 ^d^	24.15 ^a^	24.08 ^c^	19.73 ^a^	29.22 ^c^	22.40 ^a^	23.99 ^c^	25.02 ^a^	25.58 ^c^
LSD (*p* ≤ 0.05)										
Treatment	0.04	0.07	0.5	0.7	0.42	0.83	0.47	0.7	0.52	0.75
SO_2_ Level	0.06	1.02	0.71	0.99	0.59	1.17	0.67	0.98	0.74	1.06
SO_2_ Level: Treatment	0.09	1.45	1.01	1.4	0.83	1.66	0.94	1.39	0.05	1.5

Ambient = 3–5 ppb SO_2_, i.e., air without any addition of SO_2_, MI = inoculation of plants soil with 2000 J_2_ of *M. incognita* plant. Salicylic acid = (ppm), carotenoids = (µg g^−1^ fresh leaf). Figures in a column followed by different alphabets are significantly different at *p* ≤ 0.05 according to Tukey’s test.

**Table 5:** Effect of intermittent exposures of SO_2_ and root-knot nematode (M. incognita) inoculation on physiological parameters of five cucurbits.

Cucurbits	Bitter Gourd		Bottle Gourd		Sponge Gourd		Cucumber		Pumpkin	
Treatments	Transpiration	Stomatal Conductance	Transpiration	Stomatal Conductance	Transpiration	Stomatal Conductance	Transpiration	Stomatal Conductance	Transpiration	Stomatal Conductance
Ambient (Uninoculated)	1.91 ^d^	1.63 ^c^	2.31 ^c^	1.82 ^c^	2.81 ^c^	2.91 ^c^	2.82 ^c^	2.11 ^c^	2.11 ^c^	1.90 ^c^
25	2.04 ^cd^	1.75 ^bc^	2.54 ^b^	2.00 ^b^	3.07 ^b^	3.18 ^b^	3.09 ^b^	2.31 ^b^	2.33 ^b^	2.10 ^b^
50	2.09 ^bc^	1.78 ^b^	2.56 ^b^	2.01 ^b^	3.10 ^b^	3.22 ^b^	3.12 ^b^	2.33 ^b^	2.35 ^b^	2.12 ^b^
75	2.12 ^abc^	1.81 ^ab^	2.65 ^b^	2.09 ^b^	3.20 ^b^	3.31 ^b^	3.24 ^b^	2.41 ^b^	2.43 ^b^	2.19 ^b^
Ambient (Inoculated)	2.06 ^bcd^	1.77 ^b^	2.54 ^b^	2.00 ^b^	3.08 ^b^	3.18 ^b^	3.06 ^bc^	2.30 ^b^	2.33 ^bc^	2.09 ^bc^
MI + 25	2.20 ^ab^	1.88 ^ab^	2.92 ^a^	2.29 ^a^	3.54 ^a^	3.67 ^a^	3.58 ^a^	2.68 ^a^	2.70 ^a^	2.43 ^a^
MI + 50	2.25 ^a^	1.93 ^a^	3.00 ^a^	2.36 ^a^	3.60 ^a^	3.72 ^a^	3.62 ^a^	2.71 ^a^	2.77 ^a^	2.49 ^a^
MI + 75	2.26 ^a^	1.94 ^a^	3.00 ^a^	2.36 ^a^	3.60 ^a^	3.73 ^a^	3.67 ^a^	2.75 ^a^	2.79 ^a^	2.51 ^a^
LSD (*p* ≤ 0.05)										
Treatment	0.05	0.04	0.06	0.05	0.08	0.08	0.08	0.06	0.06	0.05
SO_2_ Level	0.07	0.06	0.09	0.07	0.1	0.11	0.1	0.08	0.08	0.08
SO_2_ Level: Treatment	0.1	0.08	0.13	0.10	0.15	0.16	0.16	0.11	0.12	0.1

Ambient = 3–5 ppb SO_2_, i.e., air without any addition of SO_2_, MI = inoculation of plants soil with 2000 J_2_ of *M. incognita* plant. Transpiration = (mmol H_2_O m^−2^ s^−1^), Stomatal Conductance = (mol m^−2^ s^−1^). Figures in a column followed by different alphabets are significantly different at *p* ≤ 0.05 according to Tukey’s test.

## Data Availability

Not applicable.
